# Biosecurity and Dual-Use Research: Gaining Function – But at What Cost?

**DOI:** 10.3389/fpubh.2015.00013

**Published:** 2015-02-02

**Authors:** Kathleen M. Vogel, Amanda J. Ozin, Jonathan E. Suk

**Affiliations:** ^1^North Carolina State University, Raleigh, NC, USA; ^2^European Centre for Disease Control and Prevention, Stockholm, Sweden

**Keywords:** biosecurity, dual-use research, influenza, editorial, bioterrorism

In September 2011, scientists announced new experimental findings that would not only threaten the conduct and publication of influenza research, but would have significant policy and intelligence implications. The findings presented a modified variant of the H5N1 avian influenza virus (hereafter referred to as the H5N1 virus) that was transmissible via aerosol between ferrets (1, 2). These results suggested a worrisome possibility: the existence of a new airborne and highly lethal H5N1 virus that could cause a deadly global pandemic. In response, a series of international discussions on the nature of dual-use life science arose (3). More proposed “gain-of-function (GOF)” research on the flu, and other respiratory viruses such as severe acquired respiratory syndrome (SARS) and middle east coronavirus (MERS-CoV), has led to this work being labeled as having “potential pandemic potential (PPP).”

Scientists and other interested parties are increasingly asked to more clearly state the risks and benefits of this kind of research and whether new regulations and oversight mechanisms are needed. More recently, controversies such as reported accidents and lax controls over dangerous pathogens in high profile research labs have once again raised the issue of accounting and safeguards of dangerous pathogens, with new calls for greater transparency of the oversight of these materials (4, 5). The emerging field of synthetic biology is also raising concerns about its current and future impact on human health and the environment, and its potential for bioterrorism by do-it-yourself biologists. With the Ebola outbreaks happening as we began to work this editorial, we have encountered additional (but fairly speculative!) discussion about the threat of bioterrorism during naturally occurring outbreaks and how this risk could be dealt with by the health security agenda.

Regardless of where one finds oneself on the topic, it seems clear that advances in the life sciences are creating new ethical, safety, regulatory, and security challenges. To what extent such research should be conducted, published, and governed? Who should have a say in these outcomes? What viable alternatives exist? Since 2001, there have a variety of national and global initiatives to increase biosecurity, while not unduly inhibiting responsible scientific innovation. Various countries are continuing to develop or revamp their biosecurity regimes. The traditional “bottom up” approach of scientist self-governance for biosecurity is increasingly in question, but controversial changes to the National Science Advisory Board for Biosecurity in the United States indicate that top-down approaches are also not a panacea.

This special issue was devoted to contributions that explored this matrix of issues from a variety of case study and international perspectives. This issue was a challenge to manage because of the rapidly evolving nature of developments in the field even from when we first issued the call for papers in December 2013. Emblematic of the topic itself, this made it difficult to draw a line on what to include in the issue as new submissions were entered and new controversies arose. The debate and discussion over the dual-use implications of emerging infectious diseases and the life sciences continues and will continue in the foreseeable future. We thank the authors of this special issue for an excellent set of papers to starting framing and prioritizing the national and international dialog on these timely issues.

The articles in this issue ultimately clustered around five central themes: (1) dual-use as a unique kind of policy problem; (2) involving diverse stakeholders in dual-use discussions; (3) instituting a culture of responsibility among scientists; (4) producing more evidence-based risk-benefit analyses of dual-use research; and (5) developing greater oversight, control, and standardization of dual-use procedures.

For the first theme, Rappert (6) noted that dual-use has been a largely “non-problem” – a curious phenomenon. Rappert (6) notes that although much concern has been cited with the misuse of the life sciences since September 11, there have been very few research identified as “of concern.” Moreover, Jefferson and colleagues (7) find that there has been a lot of mythmaking around synthetic biology that has been used to mobilize support, resources, and action for focusing policy attention on this field. Koblentz (8), however, finds that dual-use is an inherently “wicked problem” that makes it resistant to long-lasting solutions. In contrast, Murdock and Koepsell (9) argue that dual-use research is a classical principal-agent problem and that this kind of asymmetry between governments and scientists, creates the tensions that we see in regulation.

Connecting to the second theme, Suk et al. (10) argue that the public health sector could be brought in more to dual-use discussion to help guide policy decisions and promote actions along all phases of the research cycle. To date, the authors find that public health perspectives have been an underutilized resource in dual-use/biosecurity discussions. Kosal (11) argues more dramatically that improving public health could serve as a powerful active deterrent to those who might wish to launch a bioterrorist attack.

Laboratory biosafety and biosecurity are closely intertwined concepts, both dependent on compliance with appropriate regulations, laws, and oversight mechanisms. In both, there is no such thing as “zero risk.” As concerns biosafety, it is important to note that safety consists not simply of the design of laboratories, but also crucially in the trained people that work there, the implementation of regulations, and the use of robust risk-based approaches to mitigate adverse events. In the third theme cutting cross the papers, Jacobsen et al. (12) and Sijnesael et al. (13) argue that we need to internally cultivate responsibility within specific organizations that handle dangerous pathogens, as well as the larger life science community. These authors also argue that we need a more diverse set of stakeholders in discussions about dual-use issues and in the development and implementation of new oversight and assessment measures. Further, Jacobsen et al. (12) as well as Klotz and Sylvester (14) both argue that we need more quantitative and qualitative risk-benefit analyses for assessing research with dual-use potential. In the area of governance, Smith and Scott (15), as well as Lev and Samimian-Darash (16), Ehrlich (17), and Jacobsen et al. (12) all advocate for the need for new oversight and governance structures for research and funding of dual-use science.

In sum, what we see from these papers and continuing media coverage is that the debate of dual-use is growing, gaining more public, expert, and policy attention. But as the papers in this issue suggest, these debates need to happen at higher policy levels. Moreover, there is the need for more inculcation of scientific responsibility and norms at the local, national, and global level. Countries need to continue to work on improving their biosecurity efforts and develop some key indicators to not only show that they are committed to biosecurity and biosafety, but they are implementing, monitoring, and assessing key aspects at the local and national level. This needs to include not only academic and government research institutions, but also those in the private sector. Within this context, the underlying objective should be, ultimately, to improve and not threaten public health, but exactly how to do this is remains an outstanding and elusive question. Finally, we need more review and accounting both nationally and globally about what biosecurity measures are in place, what gaps still exist, and how to remedy these shortcomings.

## Conflict of Interest Statement

The authors declare that the research was conducted in the absence of any commercial or financial relationships that could be construed as a potential conflict of interest.

## References

[1] Herfst S Schrauwen EJ Linster M Chutinimitkul S de Wit E Munster VJ Airborne transmission of influenza A/H5N1 virus between Ferrets. Science (2012) 336(6088):1534–41. 10.1126/science.1213362 22723413 PMC4810786

[2] Imai M Watanabe T Hatta M Das SC Ozawa M Shinya K Experimental adaptation of an influenza H5 HA confers respiratory droplet transmission to a reassortant H5 HA/H1N1 virus in ferrets. Nature (2012) 486(7403):420–8. 10.1038/nature10831 22722205 PMC3388103

[3] Dual-use life science research has been defined as that which, “could be directly misapplied to pose a significant threat with broad potential consequences to public health and safety, agriculture crops, and other plants, animals, the environment, materiel, or national security”. Available from: http://www.phe.gov/s3/dualuse/Documents/durc-policy.pdf

[4] Fausset R McNeill DG Jr After Lapses, C.D.C. Admits a Lax Culture at Labs. The New York Times (July 13, 2014).

[5] *The White House Office of Science and Technology Policy. Doing Diligence to Assess the Risks and Benefits of Life Sciences Gain-of-Function Research* (2014). Available from: http://www.whitehouse.gov/blog/2014/10/17/doing-diligence-assess-risks-and-benefits-life-sciences-gain-function-research

[6] Rappert B. Why has not there been more research of concern? Front Public Health (2014) 2:74. 10.3389/fpubh.2014.00074 25101254 PMC4106452

[7] Jefferson C Lentzos F Marris C. Synthetic biology and biosecurity: challenging the “myths”. Front Public Health (2014) 2:115. 10.3389/fpubh.2014.00115 25191649 PMC4139924

[8] Koblentz GD Dual-use research as a wicked problem. Front Public Health (2014) 2:113 10.3389/fpubh.2014.00113 25140299 PMC4121526

[9] Murdock KLE Koepsell D Principals, agents, and the intersection between scientists and policy-makers: reflections on the H5N1 controversy. Front Public Health (2014) 2:109 10.3389/fpubh.2014.00109 25140298 PMC4122202

[10] Suk JE Bartels C Broberg E Struelens MJ Ozin AJ Dual-use research debates and public health: better integration would do no harm. Front Public Health (2014) 2:114 10.3389/fpubh.2014.00114 25309890 PMC4162379

[11] Kosal ME A new role for public health in bioterrorism deterrence. Front Public Health (2014) 2:278 10.3389/fpubh.2014.00278 25540775 PMC4261597

[12] Jacobsen KX Mattison K Heisz M Fry S Biosecurity in emerging life sciences technologies, a Canadian public health perspective. Front Public Health (2014) 2:198 10.3389/fpubh.2014.00198 25401097 PMC4212609

[13] Sijnesael PCC van den Berg LM Bleijs DA Odinot P de Hoog C Jansen MWJC Novel Dutch self-assessment biosecurity toolkit to identify biorisk gaps and to enhance biorisk awareness. Front Public Health (2014) 2:197 10.3389/fpubh.2014.00197 25368863 PMC4202726

[14] Klotz LC Sylvester EJ The consequences of a lab escape of a potential pandemic pathogen. Front Public Health (2014) 2:116 10.3389/fpubh.2014.00116 25157347 PMC4128296

[15] Smith FL Kamradt-Scott A Antipodal biosecurity? Oversight of dual use research in the United States and Australia. Front Public Health (2014) 2:142 10.3389/fpubh.2014.00142 25279368 PMC4165235

[16] Lev O Samimian-Darash L Biosecurity policy in the US: a critical assessment. Front Public Health (2014) 2:110 10.3389/fpubh.2014.00110 25136548 PMC4120851

[17] Ehrlich SA H5N1: a cautionary tale. Front Public Health (2014) 2:117 10.3389/fpubh.2014.00117 25161996 PMC4129514

